# Loss of control of herpes suppression following bariatric surgery

**DOI:** 10.1177/09564624241264584

**Published:** 2024-06-25

**Authors:** Shyam Samraj, Kimberley Olasoji, Raj Patel

**Affiliations:** 1 Department of Guys Campus, Kings GKT Medical School, 405987 University of London, London, UK; 2 Solent Sexual Health, Southampton, UK; 3 Faculty of Medicine, 7423 University of Southampton, Southampton, UK

**Keywords:** Herpes simplex virus, viral disease, surgery, other

## Abstract

We report a 42-year-old female with confirmed recurrent genital herpes (HSV-2), which was well controlled on suppressive antiviral therapy with aciclovir 400 mg twice daily. The patient required bariatric surgery in order to manage what was deemed a dangerously high BMI. A Roux-en-Y procedure was performed which effectively reduced her weight; however, herpes suppression become ineffective post operatively, with serious herpes related complications, despite increasing the total dose of aciclovir and the frequency from twice daily to three times a day. Complete herpes control was restored by changing therapy to valaciclovir 500 mg twice daily. The Roux-en-Y procedure is the most common form of bariatric surgery. Consequences on the efficacy of different herpes antivirals can be predicted from what is known of their properties and sites of absorption. Similar problems with herpes virus suppression may be avoided by an anticipated change in therapy preoperatively.

## Introduction

A 42-year-old HIV-negative heterosexual female was diagnosed with probable herpes simplex virus type 2 by her family doctor in 2014. At presentation she had developed bilateral ulcerated genital lesions, and on follow up she had frequent unilateral lesions following a classical timeline for recurrent genital herpes. These settled into a pattern of monthly recurrences after the first 6 months. No prodromal symptoms or obvious precipitants were identified. The patient was finding it difficult to form a physical relationship due to the constant fear of a recurrence, possible onward transmission and the soreness that recurrences entailed. She was commenced on aciclovir 400 mg twice daily by the general practitioner. On suppressive therapy her recurrences were controlled with only the mildest of breakthrough episodes (approximately once a year). In 2017 her partner was also diagnosed with genital herpes.

## Case report

From a young age, the patient had always struggled to manage her weight. Despite diet and exercise, her weight had gradually risen over time, and by 2019 the patient weighed 152 kg, with a body mass index of 65.9. She had become depressed and a referral for bariatric surgery was made in 2019. A Roux-en-Y procedure was carried out by laparoscopic surgery – the stomach size was substantially reduced, and the new smaller stomach attached directly to the jejunum, bypassing the duodenum which now formed a cul-de-sac off the jejunum. She lost 64 kg over the space of 3 years post procedure. However, almost immediately post-operatively, there was an increase in the frequency of her herpes recurrences which became as frequent as her pre-aciclovir therapy, being often extended and contingent. Following guidelines her general practitioner intensified the frequency of acyclovir to three and four times daily – to no avail. The patient was referred to the GUM clinic in October 2022 where she attended with her partner. Her history was mostly given by her partner who explained that she had had become withdrawn (virtually mute) and very depressed because of her HSV disease. She also had started expressing suicidal ideations. Causes of major immunocompromise were excluded and HSV confirmed (she was shown to be HIV-negative and was seropositive for HSV-2 antibodies) and her medication was changed from aciclovir to valacyclovir 500 mg twice daily. At a review appointment in January 2023, the patient attended (on her own) and reported there had been no herpes recurrences and an almost total reduction in her depressive symptoms. On follow up in April 2023 she had maintained these changes and was discharged from the clinic with advice to not alter or change the dose of valaciclovir.

## Discussion

Absorption of aciclovir after oral administration is slow, variable and incomplete. The bioavailability of oral aciclovir is low and decreases with increasing dosage. The average time to peak concentrations is approximately 2 h and achievable peak concentrations following oral administration are less than 6 μmol/L with oral forms at standard suppressive therapy doses.^1^ The molecule being highly polar is effectively only absorbed in the upper GI tract. Limited oral bioavailability is a feature of guanosine analogues and penciclovir (the active component of famciclovir) is even less bioavailable and oral administration (to achieve therapeutic levels) is not possible.^2^ In contrast, the presence of an l-valyl ester in valaciclovir increases its lipophilicity, allowing for greater absorption and a bioavailability 3-to-5-fold times greater than that of oral aciclovir, at around 54%, increasing its efficacy as suppressive therapy.^3^

The Roux-en-Y procedure bypasses key areas of the upper bowel where aciclovir is absorbed. The procedure is the most common form of bariatric surgery and is usually chosen when a significant and sustained reduction in weight is required. As the procedure bypasses the area where aciclovir is principally absorbed, this can result in the loss of control of clinical disease. The effect of suppressive therapy wears off completely after a therapy interruption of 5 days so these effects may become apparent in the immediate post-operative period,^4^ which occurred in this case.

With the rise in bariatric procedures being offered to patients, clinicians need to be aware of the consequences of surgery to the pharmacokinetics of medication. Certain procedures will compromise antiviral therapy and alternatives (such as valaciclovir) which do not rely on absorption from the upper bowel may be required.^5^
